# Optimal treatment occasion for ultrasound stimulated microbubbles in promoting gemcitabine delivery to VX2 tumors

**DOI:** 10.1080/10717544.2022.2115163

**Published:** 2022-09-01

**Authors:** Tingting Luo, Luhua Bai, Yi Zhang, Leidan Huang, Hui Li, Shunji Gao, Xiaoxiao Dong, Ningshan Li, Zheng Liu

**Affiliations:** a Department of Ultrasound, Xinqiao Hospital, Army Medical University, Chongqing, China; b Department of Ultrasound, The First Affiliated Hospital of Shenzhen University, Shenzhen Second People’s Hospital, Shenzhen, China; c Department of Ultrasound, General Hospital of Central Theatre Command, Wuhan, China

**Keywords:** diagnostic ultrasound, microbubbles, chemotherapy, treatment occasion, drug concentration

## Abstract

Ultrasound stimulated microbubbles (USMB) is a widely used technology that can promote chemotherapeutic delivery to tumors yet the best treatment occasion for USMB is unknown or ignored. We aimed to determine the optimal treatment occasion for USMB treatment to enhance tumor chemotherapy to achieve the highest drug concentration in tumors. Experiments were conducted on VX2 tumors implanted in 60 rabbits. Gemcitabine (GEM) was intravenously infused as a chemotherapeutic agent and USMB was administered before, during or after chemotherapy. USMB was conducted with a modified diagnostic ultrasound at 3 MHz employing short bursts (5 cycles and 0.125% duty cycle) at 0.26 MPa in combination with a lipid microbubble. Subsequently, tumor blood perfusion quantitation, drug concentration detection, and fluorescence microscopy were performed. The results showed that the group that received USMB treatment immediately after GEM infusion had the highest drug concentration in tumors, which was 2.83 times that of the control group. Fifteen tumors were then treated repeatedly with the optimal USMB-plus-GEM combination, and along with the GEM and the control groups, were studied for tumor growth, tumor cell proliferation, apoptosis, and related cytokine contents. The combined treatment significantly inhibited tumor growth and promoted apoptosis. The levels of related cytokines, including HIF-1α, decreased after six combination therapies. These results suggest that the optimal treatment occasion for USMB occurs immediately after chemotherapy and tumor hypoxia improves after multiple combination therapies.

## Introduction

Chemotherapy remains the primary clinical treatment for most solid tumors, either on its own or in combination with other therapeutic modalities (Miller et al., 2019). However, the rapid proliferation of tumor cells and structural abnormalities of tumor vasculature form a typical poor blood-perfused and hypoxic microenvironment, which prevents chemotherapeutic drugs from entering the tumor, which is one of the main reasons for therapeutic resistance (Sorace et al., 2012; Graham & Unger, 2018; Snipstad et al., 2021b). Tumor tissues also develop a high interstitial fluid pressure that limits drug penetration into the tumor, even if the leaky vasculature permits drug extravasation (Heldin et al., 2004).

For a treatment to be successful, a sufficient amount of the drug has to penetrate vascular walls and reach the interior of tumor cells (Knezevic & Clarke, 2020; Wei et al., 2021). Many attempts have been made to improve the drug delivery of chemotherapy by enhancing the blood supply of tumor like thermochemotherapy (van Rhoon et al., 2020), or trying to normalize chaotic tumor vascular networks (Itatani et al., 2018). Ultrasound (US) enhanced chemotherapy or so called “sonochemotherapy” is one potential solution (Lammertink et al., 2015b). The mechanism of sonochemotherapy is believed to be a cavitation-related sonoporation effect which increases the permeability of tumor vessels and tissues, thus facilitating local chemotherapeutic drug delivery (Lentacker et al., 2014; Lammertink et al., 2015b; Bouakaz et al., 2016; Chowdhury et al., 2020). Recently, another potential therapeutic effect, ultrasound stimulated microbubbles (USMB) stimulated tumor perfusion enhancement, had been reported and might contribute to the mechanism of improving tumor hypoxia (Snipstad et al., 2018; 2021a).

Ultrasound targeted microbubble destruction (UTMD) or USMB has been widely used in promoting chemotherapy of various experimental solid tumors (Todorova et al., 2013; Kotopoulis et al., 2014; Ingram et al., 2020; Shen et al., 2020; Xia et al., 2021). For these studies, local drug concentration and tumor growth inhibition were the primary concerns because they are directly related to major therapeutic effects in a clinical context (Kurdziel et al., 2011). A lot of sonochemotherapy studies have attempted to deliver drug using drug-loaded microbubbles (MBs) (Nesbitt et al., 2018; Gong et al., 2019). Regardless of the low-loading capacity, stability and high cavitation threshold (thick shell) of therapeutic MBs, new drug development usually takes over ten years (Gong et al., 2019). By comparison, a coadministration strategy using commercially available diagnostic US, MBs, and chemotherapeutic drugs seems to be a better approach for clinical translation. A primary pilot study on clinical pancreatic cancer demonstrated an incredible result using a combination of the three technologies (Dimcevski et al., 2016).

Unlike drug-loaded MBs, the optimal treatment occasion or the best time point for USMB participation needs to be known for the coadministration strategy. This depends on the appropriate overlap of the plasma concentration of the drug and the temporal “acoustic window” created by USMB (Tzu-Yin et al., 2013). Previous studies found the permeability of cells or tissues decreased over time after USMB treatment, so the optimal occasion for USMB should be right before chemotherapy (Marty et al., 2012; Lammertink et al., 2015a). However, other studies reported that administering USMB at the peak plasma level of the drug could obtain the best therapeutic effects (Escoffre et al., 2013; Dimcevski et al., 2016). No study has completely compared the USMB-promoted drug release to tumors at different time points before, during and after intravenous chemotherapy, which is a critical factor in sonochemotherapy.

In this study, we explored the optimal treatment occasion for USMB to cut into chemotherapy. Simultaneously, other influencing factors, including acoustic parameters, treatment time, and MB concentration, were all fixed. Five experimental groups were set according to different USMB treatment occasions to determine the highest drug concentration in the tumors. Furthermore, we selected the optimal combination for a long-term treatment experiment to verify its inhibitory effect on tumor growth.

## Materials and methods

### Animal models

A total of 75 New Zealand rabbits (male and female, weight 2–2.5 kg) were required for this experiment. All rabbits were purchased from the Animal Laboratory of Xinqiao Hospital. All animal experiments were performed in accordance with the regulations of the Laboratory Animal Welfare and Ethics Committee of the Army Medical University.

Live VX2 tumor tissue was removed from a carrier rabbit and cut into cubic pieces of approximately 1 mm³ in size. A tumor cube was implanted in each rabbit on either side of the inner thigh. It usually takes 20 days for the tumors to reach a size of approximately 1 cm in diameter. For animal anesthesia, the rabbits were intramuscularly injected with xylazine hydrochloride at 0.15 ml/kg to relax the muscle. After establishing bilateral auricular vein channels, 2% pentobarbital sodium was injected intravenously at 0.1 ml/kg. The rabbits were then fixed in the supine position. One venous channel was used for MBs injection and the other for gemcitabine (GEM) injection.

### US equipment

Tumor US imaging and USMB therapy were performed using a VINNO 70 (VINNO Technology Co. Ltd, Suzhou, China) US system with an X4-12L linear array transducer. VINNO 70 is an innovative therapeutic US device with a full diagnostic imaging function and an integrated MB cavitation regulation module for therapy, named VFlash. This specially designed VFlash module is functioned as a conventional MB flash mode during contrast-enhanced US (CEUS) imaging. It is also able to regulate MB cavitation with multiple variable acoustic parameters, including mechanical index (MI), frequency, pulse length (PL), pulse repetition frequency (PRF), and pulse/interval time. In this experiment, therapeutic US exposures were applied with the X4-12L transducer operating at the central frequency of 3 MHz. The sonication scheme comprised five cycles of PL and 1500 Hz PRF. The MI displayed on the screen was 0.25. The VFlash worked with an alternative mode of 1 s on/1 s off (pulse/interval). During USMB treatment, a rectangular region of interest (ROI) was set to cover the tumor. The therapeutic beams were designated to be focused within the ROI using electronic focusing technology. The peak negative pressure (PNP) within a ROI of 1 × 1 cm was measured using a membrane hydrophone (HMB-0500, ONDA Corp., Sunnyvale, CA, USA) positioned 2 cm away from the transducer surface. The transducer was placed above the hydrophone and separated using degassed water in a sink (AIMS III, ONDA Corp.). The PNP was determined to be 0.26 MPa when the MI was set to 0.25, so the actual MI should be 0.15 (Boissenot et al., 2016).

### MBs

A lipid-coated MB with a perfluoropropane gas core called Zhifuxian was used for both the CEUS and USMB treatment. The MBs were prepared according to previously described protocols (Liu et al., 2011). The average particle diameter of the MBs was approximately 2.1 μm, and the concentration of the MBs suspension was 4–9 × 10^9^/ml (Liu et al., 2011). For CEUS, 0.1 ml of MBs suspension was bolus injected. For USMB treatment, 0.1 ml of MBs suspension was diluted to 3 ml with saline. An initial 0.5 ml of the diluted suspension was injected to fill the catheter at the beginning, followed by slow injection of 0.05 ml suspension every 30 s for 20 min.

### Treatment protocols

**Figure 1.**
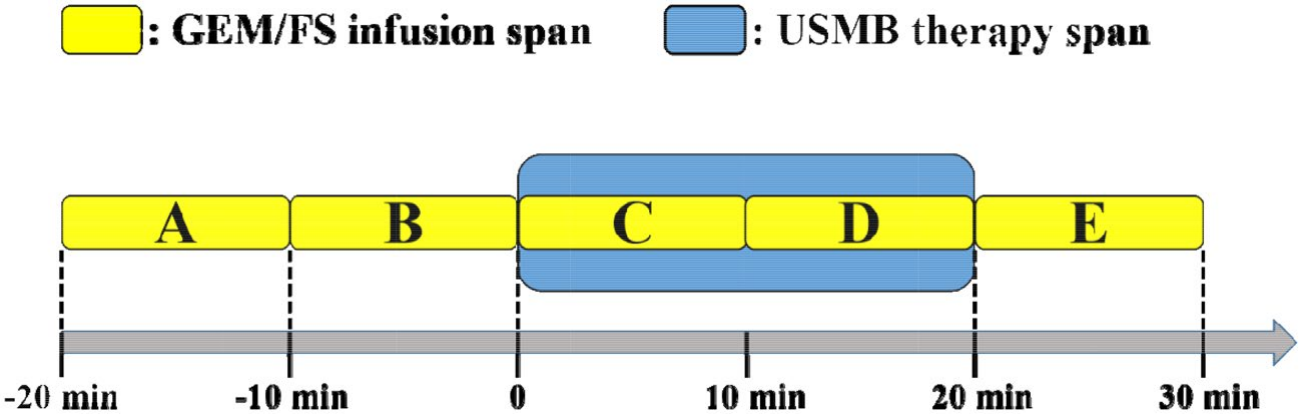
Treatment procedure flow chart with time span of chemotherapy and USMB treatment in the five experimental groups.

Two types of experiments were conducted. In Experiment Set 1, experiments were conducted on 60 tumor-bearing rabbits to determine the optimal occasion for USMB treatment (Figure 1). Five experimental groups (A-E) were assigned according to different USMB treatment occasions, and one control group (F) was set. Within each group, eight animals received intravenous injection of GEM (25 mg/kg) for 10 min, while two others received intravenous injection of fluorescein sodium (FS, 3 mg/kg). Groups A and B started GEM/FS injection 20 min and 10 min before USMB treatment, respectively; groups C and D received GEM/FS injection during the first half (10 min) and the second half (10 min) of USMB treatment, respectively; group E received GEM/FS injection immediately after USMB treatment. Group F, which received GEM/FS and MBs only without US exposure, served as the control. USMB treatment lasted for 20 min, with the fixed parameters described above. CEUS was performed at a low MI (0.11 on the screen) before and immediately after USMB treatment to examine tumor blood perfusion, and the dynamic images were stored for 60 s for further analysis. All animals were sacrificed by air injection via the auricular vein 40 min after GEM/FS infusion to obtain tumor tissues for high-performance liquid chromatography (HPLC) and fluorescence microscopy.

In Experiment Set 2, the long-term therapeutic effects of sonochemotherapy were assessed. Fifteen tumor-bearing rabbits were randomly separated into three groups: USMB + GEM group (*n* = 5), GEM group (*n* = 5), and control group (*n* = 5). According to the clinical medication course of GEM, the animals in the USMB + GEM and GEM groups were treated with GEM (25 mg/kg) once a week for a total of six treatments, with one-week intervals after every three weeks of treatment (Shibata et al., 2016). The tumors of the USMB + GEM group were again treated with USMB immediately after GEM infusion like group B of Experiment Set 1, while animals in the control group were administered only 3 ml of saline infusion. All tumors were measured using two-dimensional (2 D) imaging of VINNO 70 with an X4-12L transducer once a week, just before every treatment. All animals were euthanized by air injection via the auricular vein 24 h after the last treatment. The tumors were harvested for Ki67 staining for cell proliferation analysis, terminal deoxynucleotidyl transferase-mediated dUTP nick-end labeling (TUNEL) staining for apoptosis analysis, and enzyme-linked immunosorbent assay (ELISA) for cytokine detection.

### CEUS quantitation and tumor perfusion rate calculation

For CEUS quantitation, dynamic image clips of CEUS were analyzed using the internal analysis software of VINNO 70. First, a 60 s video clip was marked from the beginning. Second, boundary of the tumor was manually traced at the peak intensity (PI) of the arterial phase. Then the software could automatically generate a time-intensity curve (TIC) of the drawn area and the values of the PI and area under curve (AUC). The PI is the highest point of the TIC, and the AUC is the integration of the area under the TIC within 60 s (Lee et al., 2019).

For the calculation of tumor perfusion area rate, images of the peak contrast intensity were captured in the CEUS video. Then, the entire tumor area and tumor perfused area of the images were manually delineated and computed using Image J 1.8.0 software (National Institutes of Health, Bethesda, MD, USA). The tumor perfusion area rate was calculated as perfusion area/entire tumor area × 100%.

### HPLC

The tumors of 48 rabbits in Experiment Set 1 were collected 40 min after GEM infusion. The GEM sample was dissolved in methanol to a concentration of 2 mg/ml, and then a 5-point standard curve was drawn. Chromatogram acquisition and integration were processed using Xcalibur 3.0 software (ThermoFisher Scientific Inc., Waltham, MA, USA). The tumors were weighed and carefully cleaned. Then Zirconia grinding beads and 1 ml of methanol were added to the tumor tissue and ground for 10 min. The tissue was centrifuged at 4 °C for 10 min (13000 rpm). After centrifugation, the liquid was filtered through a 0.22-μm membrane to obtain the filtrate. The filtrate was analyzed using an UltiMate 3000 RS HPLC device (ThermoFisher Scientific Inc.). The AUC was calculated using the software, and the concentration of GEM in the samples was determined by comparing with the standard curve.

### Tumor volume quantitation and tumor growth rate

Tumor volume was calculated using three vertical 2 D US axes (length, width, and height) following the equation:

Volume = Length×Width×Height×π/6

The tumor growth rate was calculated according to the following equation:

$$\text{Tumor Growth Rate }=(V_{\text{1}}-V_{0})/V_{0}\times \text{1}00\%$$

(*V*_1_: tumor volume in the 3rd or 7th week; *V*_0_: tumor volume before the first treatment)

### Immunofluorescence staining

The tumors of 12 rabbits in Experiment Set 1 were collected 40 min after FS infusion and made into 3-μm paraffin sections. The tumor cell nuclei were stained with DAPI. The sections were examined under a fluorescence microscope.

The tumors of nine rabbits in Experiment Set 2 were collected 24 h after the last treatment and made into 3-μm paraffin sections, with three in each group. The samples were incubated with rabbit anti-Ki67 antibody (1:200, Servicebio, Wuhan, China) overnight in a wet box at 4 °C. The samples were washed with PBS for three times, then incubated with Cy3-conjugated anti-IgG (1:400, Servicebio) for 50 min at 37 °C. The cell nuclei were stained with DAPI for 10 min. The sections were then observed under a confocal laser microscope. The rate of Ki67 positive cells was calculated using Aipathwell software (Servicebio) after scanning the full slide.

### Immunohistochemical staining

The tumors of nine rabbits in Experiment Set 2 were collected 24 h after the last treatment, with three in each group. The removed tumor tissues were fixed with 4% paraformaldehyde for 30 min at 37 °C and cut into 3-μm paraffin sections. The sections were then deparaffinized and dehydrated. Apoptosis of the tumor cells was determined by TUNEL staining with an In Situ Cell Death Detection Kit (POD, Roche, Germany). The mean integrated optical density (IOD) of two random fields in each section was calculated using Image Pro Plus 6.0 software (Media Cybernetics, Silver Spring, MD, USA).

### ELISA

The tumors of 15 rabbits in Experiment Set 2 were collected 24 h after the last treatment. The tumors were cleaned with precooled PBS (pH 7.2–7.4), weighed to 1 g, and cut into pieces. Nine milliliters of PBS was added to the cut tumors, and the samples were ground on ice and centrifuged for 20 min (3000 rpm). The supernatant was collected to detect the contents of HIF-1α, VEGF, TNF-α, and TGF-β using ELISA Kits (Ruixin Biotech, Fujian, China).

### Statistical analysis

All data are presented as the mean ± standard deviation (SD). All statistical analyses were performed using SPSS 26.0 software (SPSS, Inc., Chicago, IL, USA). Statistical significance was set at *p* < 0.05. Paired t-test was used to compare pre- and post-treatment values. One-way analysis of variance was performed for the comparison of multiple groups, and the least significant difference method was used for further comparisons between the two groups.

## Results

### Tumor perfusion

**Figure 2.**
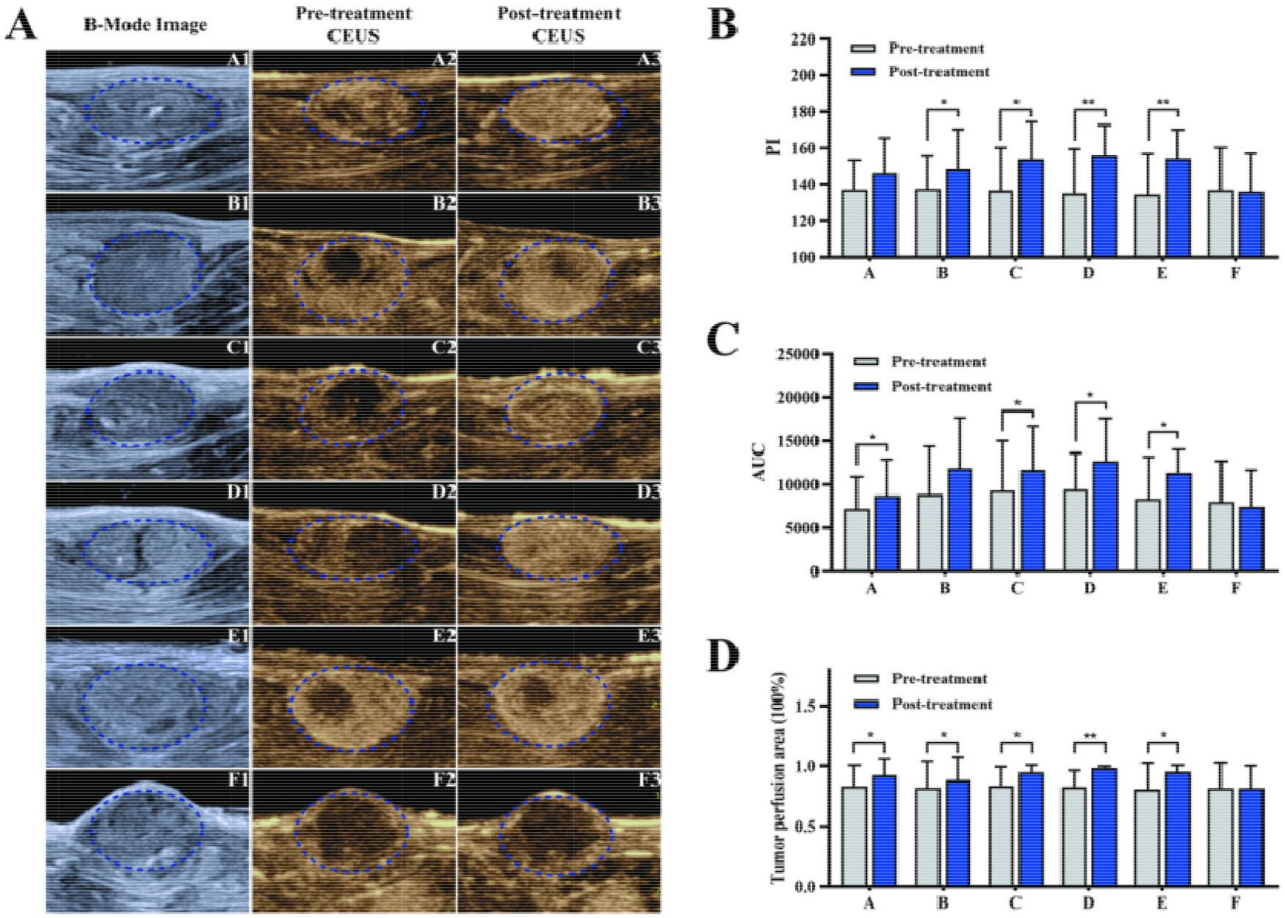
The comparison of tumor blood perfusion before and after treatment in Experiment Set 1. (A) B-Mode and CEUS images of tumors in the six groups. Tumor perfusion was improved by USMB in the five treatment groups. (B, C) Results of CEUS quantitation. The PI and AUC values of tumors in the six groups before and after treatment. The data are presented as the mean ± SD. *p < 0.05, ** *p* < 0.01. *n* = 10. (D) The perfusion area rate of tumors in the six groups before and after treatment. The data are presented as the mean ± SD. *p < 0.05, ** *p* < 0.01. *n* = 10.

Tumor blood perfusion improved both visually and quantitatively after USMB administration in all treatment groups (Figure 2 and Table 1). The PI values in the five experimental groups increased by 11.7% ± 3.6%, with the highest increase of 15.5% observed in group D. The elevation in PI values was not significant after USMB treatment in group A (*p* = 0.057). The AUC values in the experimental groups increased by 28.3% ± 6.4%, with the highest increase of 35.5% in group E. There was no significant difference in the AUC values between pre- and post-treatment in group B (*p* = 0.070). The tumor perfusion area rate increased by 13.4% ± 4.5% with the highest increase of 18.5% in group D. In the control group, there were no significant changes in the PI or AUC values, nor in the tumor perfusion area rate after treatment (*p* > 0.05).

**Table 1.** PI values, AUC values and perfusion area rate of tumors in Experiment Set 1 before treatment and after treatment ($\bar{x}$ ± *s*).

Groups	PI (dB)		AUC (dB•s)		Perfusion Area Rate (%)	
	Pre-treatment	Post-treatment	Pre-treatment	Post-treatment	Pre-treatment	Post-treatment
A	136.7 ± 16.1	146.3 ± 19.4	7233.1 ± 3668.9	8753.1 ± 4121.2*	83.3 ± 17.3	92.4 ± 13.7*
B	136.7 ± 17.6	149.3 ± 21.8*	9195.9 ± 5844.5	11561.4 ± 5640.3	82.3 ± 21.7	88.5 ± 19.1*
C	136.5 ± 23.6	153.4 ± 20.9*	9331.1 ± 5673.3	11630.1 ± 5081.2*	83.8 ± 15.4	94.6 ± 6.1*
D	134.8 ± 24.6	155.7 ± 17.0**	9444.1 ± 4140.7	12690.7 ± 4953.9*	82.9 ± 13.2	98.2 ± 1.5**
E	134.2 ± 22.5	153.8 ± 16.4**	8376.2 ± 4794.7	11353.3 ± 2669.5*	81.1 ± 21.3	95.0 ± 5.7*
F	136.6 ± 23.6	135.7 ± 21.1	8032.9 ± 4648.2	7481.3 ± 4150.7	81.9 ± 20.7	81.8 ± 18.3

^*^ *p* < 0.05 versus pre-treatment.

^**^ *p* < 0.01 versus pre-treatment.

### GEM concentration and FS distribution

**Figure 3.**
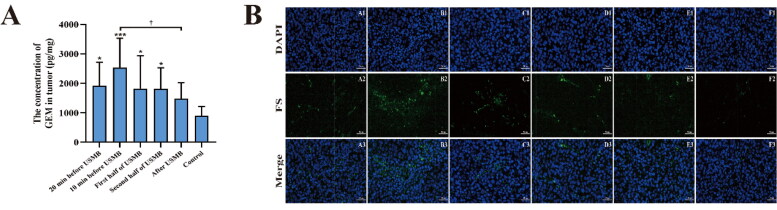
(A) The concentration of GEM in the tumors of the six groups 40 min after infusion. The data are presented as the mean ± SD. * *p* < 0.05, *** *p* < 0.001 versus the control group. † *p* < 0.05. *n* = 8. (B) Representative fluorescence images of the FS distribution in the six groups (scale bar: 50 μm).

The GEM concentrations in the tumors of groups A–F were 1919.5 ± 798.5, 2541.0 ± 994.0, 1826.4 ± 1111.1, 1815.6 ± 717.2, 1479.3 ± 545.9 and 898.0 ± 316.9 pg/mg, respectively (Figure 3A). The concentrations in the five experimental groups were all higher than those in group F, while there was no significant difference between groups E and F (*p* = 0.15). The highest GEM concentration occurred in group B, which was 2.83-fold that of group F. Furthermore, the GEM concentration in group B was significantly higher than that in group E (*p* = 0.011).

The results of FS distribution were consistent with the GEM concentrations (Figure 3B). The fluorescence images showed that the FS distribution in group B was visually more widespread and intense than that in the other groups.

### Tumor growth inhibition

**Figure 4.**
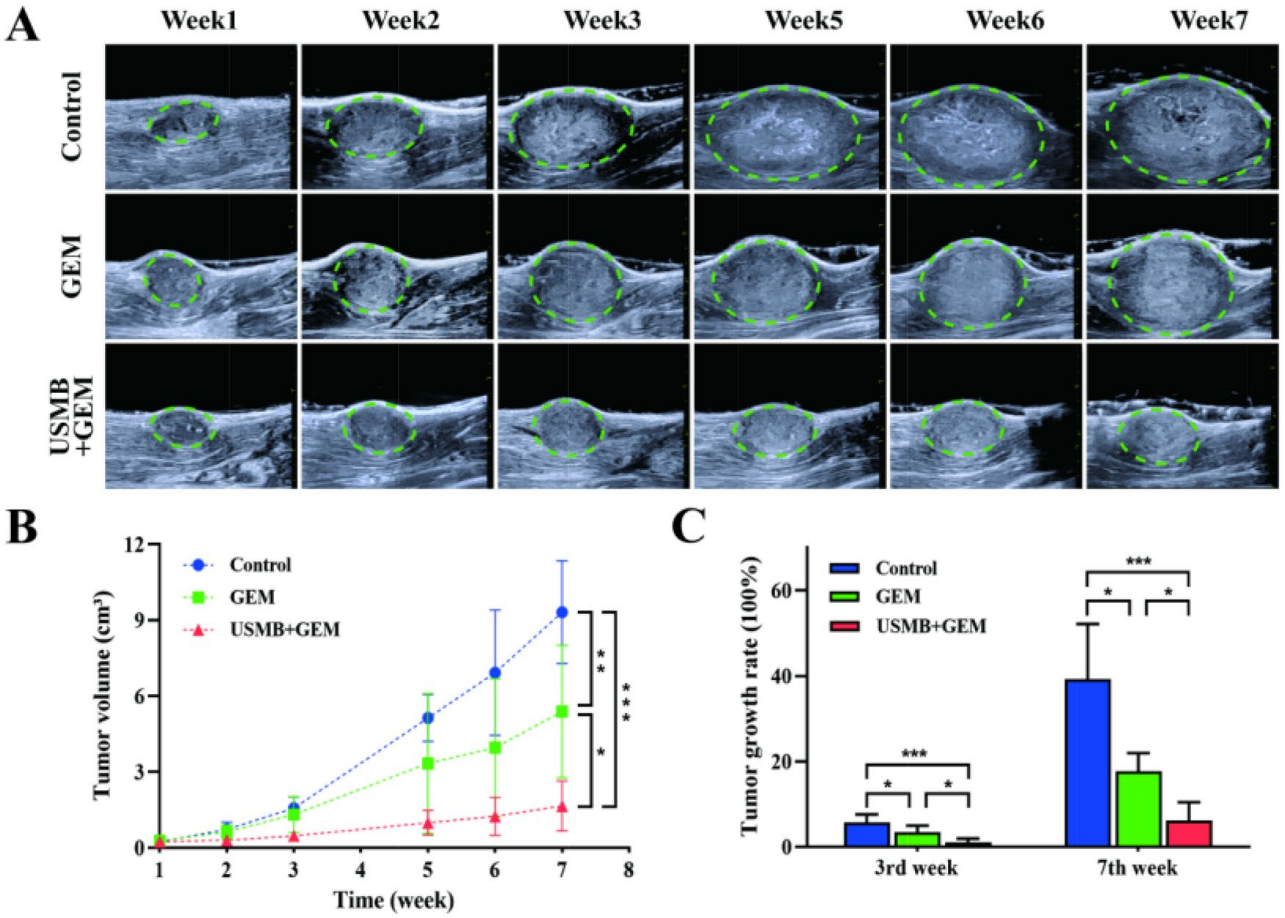
Tumor growth of the three groups in Experiment Set 2. The combination therapy showed more effective inhibitory effects on tumor growth. (A) B-Mode images of tumors in the three groups before every treatment. (B) Tumor growth curve of the three groups. The data are presented as the mean ± SD. * *p* < 0.05, ** *p* < 0.01, *** *p* < 0.001. *n* = 5. (C) Tumor growth rate of the three groups before the third and sixth treatments. The data are presented as the mean ± SD. * *p* < 0.05, *** *p* < 0.001. *n* = 5.

In Experiment Set 2, there was no significant difference in tumor volume before the first treatment among the three groups. Tumors in the USMB + GEM group grew significantly slower than those in the other two groups, and the tumors in the control group grew the fastest (Figure 4). Right before the third treatment (3rd week), the tumor growth rates of the three groups were 566.2% ± 187.5%, 340.8% ± 150.2% and 103.1% ± 88.6%, respectively. Immediately before the sixth treatment (7th week), the tumor growth rates of the three groups increased to 3922.5% ± 1288.0%, 1763.8% ± 428.8% and 615.3% ± 423.7%, respectively (Figure 4C).

**Figure 5.**
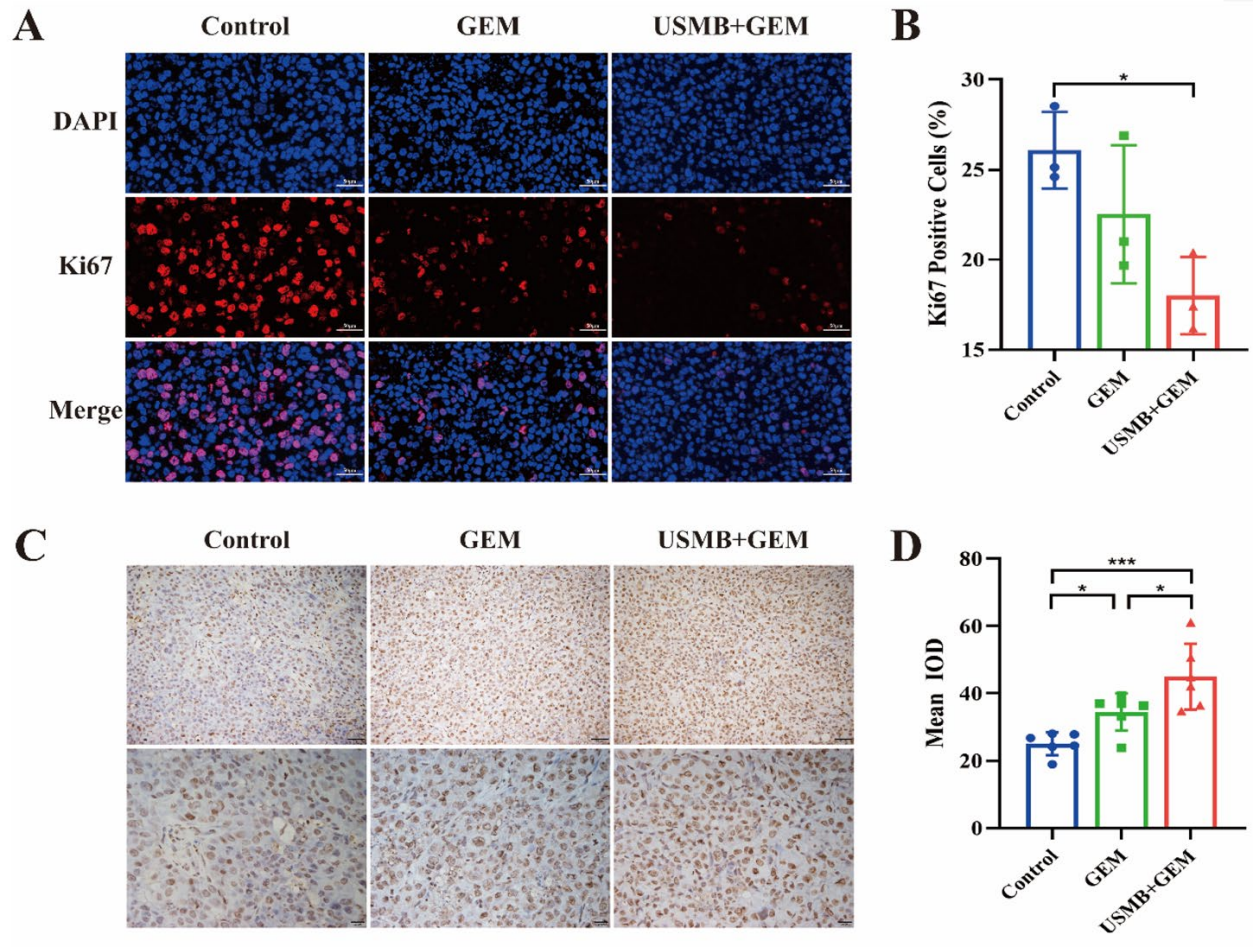
(A) Representative immunofluorescence images of tumor Ki67 expression in the three groups in Experiment Set 2 after the last treatment (scale bar: 50 μm). (B) The rate of Ki67 positive cells in the three groups. The data are presented as the mean ± SD. * *p* < 0.05. *n* = 3. (C) Representative TUNEL images of tumor cell apoptosis in the three groups in Experiment Set 2 (scale bar: 50 and 20 μm). (D) Mean IOD of TUNEL staining in the three groups. The data are presented as the mean ± SD. * *p* < 0.05, *** *p* < 0.001. *n* = 6.

Immunofluorescence staining showed that the expression of Ki67 was lower in the USMB + GEM group than in the other two groups (Figure 5A), which was consistent with the tumor growth rates. There was a significant difference in the Ki67 positive rate between the USMB + GEM and control groups (Figure 5B). TUNEL staining showed that combination therapy caused significantly more apoptosis of tumor cells in the USMB + GEM group than in the GEM and control groups (Figure 5C). There were significant differences in the mean IOD among the three groups (Figure 5D).

### Cytokines detection

The contents of several cytokines, including HIF-1α, VEGF, TNF-α, and TGF-β, were determined using ELISA. The results of ELISA are presented in Table 2. After six treatments, the contents of HIF-1α, VEGF, TNF-α, and TGF-β in the USMB + GEM group were all lower than those in the other two groups, while the control group had the highest contents.

**Table 2.** The contents of HIF-1α, VEGF, TNF-α, and TGF-β in tumors of the three groups in Experiment Set 2 ($\bar{x}$ ± *s*).

Groups	HIF-1α (pg/mg)		VEGF (pg/mg)		TNF-α (pg/mg)		TGF-β (pg/mg)
Control	6.6 ± 0.5^†***^		49.1 ± 7.1^††***^		31.1 ± 3.5^†*^		24.8 ± 3.6^††**^
GEM	5.4 ± 0.7*		38.1 ± 3.2*		26.0 ± 2.4		18.7 ± 2.0
USMB + GEM	4.3 ± 0.8^†^		29.5 ± 4.9^†^		25.8 ± 2.9		16.9 ± 2.9

† *p* < 0.05 versus GEM group.

†† *p* < 0.01 versus GEM group.

* *p* < 0.05 versus USMB + GEM group.

** *p* < 0.01 versus USMB + GEM group.

*** *p* < 0.001 versus USMB + GEM group.

## Discussion

The effect of USMB treatment on enhancing tumor chemotherapy has been verified in previous studies (Sennoga et al., 2017; Xia et al., 2021). Nevertheless challenges remain in the translation of USMB treatment from basic research to clinical practice (Sennoga et al., 2017). The treatment occasion for USMB treatment is one of the key points affecting the synergistic therapeutic efficacy. The results of this study identified the optimal treatment occasion for USMB treatment in combination with intravenous chemotherapy. In Experiment Set 1, the tumors treated with USMB immediately after GEM infusion had the highest drug concentration, which was 2.83-fold that of the control. The other three groups that received drug infusion 20 min before or during USMB treatment had similar but lower drug concentrations. The tumors that received the drug after USMB treatment had the lowest drug concentration compared with the other experimental groups, although it still was 1.65-fold that of the control. These results indicate that the USMB treatment occasion is probably an independent factor influencing drug delivery to the tumor when performing sonochemotherapy, and the optimal occasion for USMB administration is immediately after chemotherapeutic infusion. It is known that the peak plasma concentration of intravenous chemotherapy usually occurs immediately after drug infusion (Tham et al., 2008). Sometimes a delayed peak plasma level may occur when the prodrug needs to be converted into its active form (Escoffre et al., 2013). However, sonoporation always takes effect simultaneously with the onset of USMB and may last a few hours after sonication (Lammertink et al., 2015a; Yang et al., 2020). Therefore, it is in keeping with that USMB treatment occasion immediately after intravenous drug administration results in the highest local GEM release. It is also understood that other treatment occasions fail to meet the peak plasma level of the drug, so the opportunity is missed to maximize drug delivery to the tumor. Some previous studies did choose to perform sonochemotherapy immediately after drug administration and obtained noteworthy therapeutic effects (Kotopoulis et al., 2014; Zhang et al., 2021). This coadministration regimen was used in the first clinical study of pancreatic cancer and significantly extended the survival time of patients (Dimcevski et al., 2016).

In Experiment Set 1, the perfusion enhancement effect of tumors stimulated by USMB treatment was repeatedly confirmed as in previous studies (Feng et al., 2021; Li et al., 2021). The PI values of tumor CEUS increased by 11.7% ± 3.6%, the AUC values increased by 28.3% ± 6.4%, and the tumor perfusion area rate increased by 13.4% ± 4.5% in the five experimental groups. No obvious changes in tumor perfusion were observed in the control. USMB treatment was administered using a modified diagnostic US system and lipid-coated MBs. It was remarkably observed that the perfusion enhancement effect could be induced at such low parameters (MI 0.25, PL 5 cycles, and PRF 1.5 kHz). Although the perfusion enhancement effect is supposed to be stimulated concomitantly with sonoporation, it has been ignored in most previous studies (Shapiro et al., 2016). Theoretically, tumor perfusion enhancement may improve the characteristic poor-perfused and hypoxic microenvironment in central solid tumors, which is a major cause of chemotherapeutic resistance (Carmona-Bozo et al., 2021).

Based on the results of Experiment Set 1, the optimal combination of USMB and GEM as in group B, was chosen for long-term therapy and showed significant inhibition of tumor growth. The growth rate of tumors treated with this combination therapy was 615.3% ± 423.7% after seven weeks, which was almost one-third of the tumors treated with chemotherapy alone. Furthermore, the expression of HIF-1α, VEGF, TNF-α, and TGF-β in tumors was significantly inhibited after six combined treatments when compared with the other two groups. HIF-1α and VEGF play an essential role in angiogenesis and the formation of hypoxic microenvironment inside tumors (Ramjiawan et al., 2017). The contents of HIF-1α and VEGF decreased by 34.8% and 39.9%, respectively, compared with the control, suggesting that tumor angiogenesis was inhibited and the hypoxic microenvironment of tumors may be improved by combination therapy (Siveen et al., 2017; Graham & Unger 2018). TNF plays a complicated role in the development of tumors. It could promote tumor proliferation, metastasis, and angiogenesis while also inducing tumor cell death (Pusuluri et al., 2019). Previous studies have shown that the serum level of TNF-α in cancer patients decreased significantly after chemotherapy (Berberoglu et al., 2004; Michalaki et al., 2004). TGF-β has been reported to stimulate tumor invasion and metastasis (Tian et al., 2011). Therefore, the decline of TNF-α and TGF-β can indicate an effective therapeutic response.

The coadministration of diagnostic US, commercial MBs, and intravenous chemotherapy is an effective combination because it can overcome the challenges of drug-loaded MBs, such as low drug-loading and long and high expense development (Gong et al., 2019). As described above, the therapeutic benefit of drug delivery using USMB relies on enhancing drug accumulation in tumor cells or tissues (Li et al., 2012; Burke et al., 2014). For the coadministration strategy, treatment occasion or time point might be an independent influence for local drug accumulation. In this study, we concluded that USMB administration immediately after chemotherapeutic infusion could be the optimal treatment regimen for the sake of maximal drug delivery, at least for intravenous GEM infusion.

There are several limitations in this study. First, we did not compare the therapeutic effect of different USMB treatment occasions in long-term experiments. Second, the improvement in tumor hypoxia by USMB-enhanced perfusion remains a theory which requires further investigation.

## References

[CIT0001] Berberoglu U, Yildirim E, Celen O. (2004). Serum levels of tumor necrosis factor alpha correlate with response to neoadjuvant chemotherapy in locally advanced breast cancer. Int J Biol Markers 19:130–4. 15255545 10.1177/172460080401900207

[CIT0002] Boissenot T, Bordat A, Fattal E, et al. (2016). Ultrasound-triggered drug delivery for cancer treatment using drug delivery systems: From theoretical considerations to practical applications. J Control Release 241:144–63. 27667179 10.1016/j.jconrel.2016.09.026

[CIT0003] Bouakaz A, Zeghimi A, Doinikov AA. (2016). Sonoporation: Concept and mechanisms. Adv Exp Med Biol 880:175–89. 26486338 10.1007/978-3-319-22536-4_10

[CIT0004] Burke CW, Alexander E. t, Timbie K, et al. (2014). Ultrasound-activated agents comprised of 5FU-bearing nanoparticles bonded to microbubbles inhibit solid tumor growth and improve survival. Mol Ther 22:321–8. 24172867 10.1038/mt.2013.259 PMC3916048

[CIT0005] Carmona-Bozo JC, Manavaki R, Woitek R, et al. (2021). Hypoxia and perfusion in breast cancer: simultaneous assessment using PET/MR imaging. Eur Radiol 31:333–44. 32725330 10.1007/s00330-020-07067-2 PMC7755870

[CIT0006] Chowdhury SM, Abou-Elkacem L, Lee T, et al. (2020). Ultrasound and microbubble mediated therapeutic delivery: Underlying mechanisms and future outlook. J Control Release 326:75–90. 32554041 10.1016/j.jconrel.2020.06.008

[CIT0007] Dimcevski G, Kotopoulis S, Bjånes T, et al. (2016). A human clinical trial using ultrasound and microbubbles to enhance gemcitabine treatment of inoperable pancreatic cancer. J Control Release 243:172–81. 27744037 10.1016/j.jconrel.2016.10.007

[CIT0008] Escoffre JM, Novell A, Serrière S, et al. (2013). Irinotecan delivery by microbubble-assisted ultrasound: in vitro validation and a pilot preclinical study. Mol Pharm 10:2667–75. 23675982 10.1021/mp400081b

[CIT0009] Feng S, Qiao W, Tang J, et al. (2021). Chemotherapy augmentation using low-intensity ultrasound combined with microbubbles with different mechanical indexes in a pancreatic cancer model. Ultrasound Med Biol 47:3221–30. 34362582 10.1016/j.ultrasmedbio.2021.07.004

[CIT0010] Gong Q, Gao X, Liu W, et al. (2019). Drug-loaded microbubbles combined with ultrasound for thrombolysis and malignant tumor therapy. Biomed Res Int 2019:6792465. 31662987 10.1155/2019/6792465 PMC6791276

[CIT0011] Graham K, Unger E. (2018). Overcoming tumor hypoxia as a barrier to radiotherapy, chemotherapy and immunotherapy in cancer treatment. Int J Nanomedicine 13:6049–58. 30323592 10.2147/IJN.S140462 PMC6177375

[CIT0012] Heldin CH, Rubin K, Pietras K, et al. (2004). High interstitial fluid pressure - an obstacle in cancer therapy. Nat Rev Cancer 4:806–13. 15510161 10.1038/nrc1456

[CIT0013] Ingram N, McVeigh LE, Abou-Saleh RH, et al. (2020). Ultrasound-triggered therapeutic microbubbles enhance the efficacy of cytotoxic drugs by increasing circulation and tumor drug accumulation and limiting bioavailability and toxicity in normal tissues. Theranostics 10:10973–92. 33042265 10.7150/thno.49670 PMC7532679

[CIT0014] Itatani Y, Kawada K, Yamamoto T, et al. (2018). Resistance to anti-angiogenic therapy in cancer-alterations to anti-VEGF pathway. Int J Mol Sci 19:1232. 10.3390/ijms19041232 PMC5979390 29670046

[CIT0015] Knezevic CE, Clarke W. (2020). Cancer chemotherapy: The case for therapeutic drug monitoring. Ther Drug Monit 42:6–19. 31568180 10.1097/FTD.0000000000000701

[CIT0016] Kotopoulis S, Delalande A, Popa M, et al. (2014). Sonoporation-enhanced chemotherapy significantly reduces primary tumour burden in an orthotopic pancreatic cancer xenograft. Mol Imaging Biol 16:53–62. 23877869 10.1007/s11307-013-0672-5

[CIT0017] Kurdziel KA, Kalen JD, Hirsch JI, et al. (2011). Human dosimetry and preliminary tumor distribution of 18F-fluoropaclitaxel in healthy volunteers and newly diagnosed breast cancer patients using PET/CT. J Nucl Med 52:1339–45. 21849404 10.2967/jnumed.111.091587 PMC3224978

[CIT0018] Lammertink BH, Bos C, Deckers R, et al. (2015b). Sonochemotherapy: from bench to bedside. Front Pharmacol 6:138. 26217226 10.3389/fphar.2015.00138 PMC4498442

[CIT0019] Lammertink B, Deckers R, Storm G, et al. (2015a). Duration of ultrasound-mediated enhanced plasma membrane permeability. Int J Pharm 482:92–8. 25497443 10.1016/j.ijpharm.2014.12.013

[CIT0020] Lee SC, Tchelepi H, Grant E, et al. (2019). Contrast-enhanced ultrasound imaging of breast masses: Adjunct tool to decrease the number of false-positive biopsy results. J Ultrasound Med 38:2259–73. 30597640 10.1002/jum.14917 PMC7735954

[CIT0021] Lentacker I, De Cock I, Deckers R, et al. (2014). Understanding ultrasound induced sonoporation: definitions and underlying mechanisms. Adv Drug Deliv Rev 72:49–64. 24270006 10.1016/j.addr.2013.11.008

[CIT0022] Li N, Tang J, Yang J, et al. (2021). Tumor perfusion enhancement by ultrasound stimulated microbubbles potentiates PD-L1 blockade of MC38 colon cancer in mice. Cancer Lett 498:121–9. 33129956 10.1016/j.canlet.2020.10.046

[CIT0023] Li P, Zheng Y, Ran H, et al. (2012). Ultrasound triggered drug release from 10-hydroxycamptothecin-loaded phospholipid microbubbles for targeted tumor therapy in mice. J Control Release 162:349–54. 22800580 10.1016/j.jconrel.2012.07.009

[CIT0024] Liu P, Wang X, Zhou S, et al. (2011). Effects of a novel ultrasound contrast agent with long persistence on right ventricular pressure: Comparison with SonoVue. Ultrasonics 51:210–4. 20825961 10.1016/j.ultras.2010.07.008

[CIT0025] Marty B, Larrat B, Van Landeghem M, et al. (2012). Dynamic study of blood-brain barrier closure after its disruption using ultrasound: a quantitative analysis. J Cereb Blood Flow Metab 32:1948–58. 22805875 10.1038/jcbfm.2012.100 PMC3463875

[CIT0026] Michalaki V, Syrigos K, Charles P, et al. (2004). Serum levels of IL-6 and TNF-alpha correlate with clinicopathological features and patient survival in patients with prostate cancer. Br J Cancer 90:2312–6. 15150588 10.1038/sj.bjc.6601814 PMC2409519

[CIT0027] Miller KD, Nogueira L, Mariotto AB, et al. (2019). Cancer treatment and survivorship statistics, 2019. CA A Cancer J Clin 69:363–85. 10.3322/caac.21565 31184787

[CIT0028] Nesbitt H, Sheng Y, Kamila S, et al. (2018). Gemcitabine loaded microbubbles for targeted chemo-sonodynamic therapy of pancreatic cancer. J Control Release 279:8–16. 29653222 10.1016/j.jconrel.2018.04.018

[CIT0029] Pusuluri A, Wu D, Mitragotri S. (2019). Immunological consequences of chemotherapy: Single drugs, combination therapies and nanoparticle-based treatments. J Control Release 305:130–54. 31004668 10.1016/j.jconrel.2019.04.020

[CIT0030] Ramjiawan RR, Griffioen AW, Duda DG. (2017). Anti-angiogenesis for cancer revisited: Is there a role for combinations with immunotherapy? Angiogenesis 20:185–204. 28361267 10.1007/s10456-017-9552-y PMC5439974

[CIT0031] Sennoga CA, Kanbar E, Auboire L, et al. (2017). Microbubble-mediated ultrasound drug-delivery and therapeutic monitoring. Expert Opin Drug Deliv 14:1031–43. 27892760 10.1080/17425247.2017.1266328

[CIT0032] Shapiro G, Wong AW, Bez M, et al. (2016). Multiparameter evaluation of in vivo gene delivery using ultrasound-guided, microbubble-enhanced sonoporation. J Control Release 223:157–64. 26682505 10.1016/j.jconrel.2015.12.001 PMC4724495

[CIT0033] Shen Z, Shao J, Zhang J, et al. (2020). Ultrasound cavitation enhanced chemotherapy: In vivo research and clinical application. Exp Biol Med (Maywood) 245:1200–12. 32567346 10.1177/1535370220936150 PMC7437381

[CIT0034] Shibata T, Ebata T, Fujita K, et al. (2016). Optimal dose of gemcitabine for the treatment of biliary tract or pancreatic cancer in patients with liver dysfunction. Cancer Sci 107:168–72. 26595259 10.1111/cas.12851 PMC4768397

[CIT0035] Siveen KS, Prabhu K, Krishnankutty R, et al. (2017). Vascular Endothelial Growth Factor (VEGF) signaling in tumour vascularization: Potential and challenges. Curr Vasc Pharmacol 15:339–51. 28056756 10.2174/1570161115666170105124038

[CIT0036] Snipstad S, Mørch Ý, Sulheim E, et al. (2021a). Sonopermeation enhances uptake and therapeutic effect of free and encapsulated cabazitaxel. Ultrasound Med Biol 47:1319–33. 33549379 10.1016/j.ultrasmedbio.2020.12.026

[CIT0037] Snipstad S, Sulheim E, de Lange Davies C, et al. (2018). Sonopermeation to improve drug delivery to tumors: from fundamental understanding to clinical translation. Expert Opin Drug Deliv 15:1249–61. 30415585 10.1080/17425247.2018.1547279

[CIT0038] Snipstad S, Vikedal K, Maardalen M, et al. (2021b). Ultrasound and microbubbles to beat barriers in tumors: Improving delivery of nanomedicine. Adv Drug Deliv Rev 177:113847. 34182018 10.1016/j.addr.2021.113847

[CIT0039] Sorace AG, Warram JM, Umphrey H, et al. (2012). Microbubble-mediated ultrasonic techniques for improved chemotherapeutic delivery in cancer. J Drug Target 20:43–54. 21981609 10.3109/1061186X.2011.622397 PMC3417245

[CIT0040] Tham LS, Wang LZ, Soo RA, et al. (2008). Does saturable formation of gemcitabine triphosphate occur in patients? Cancer Chemother Pharmacol 63:55–64. 18305939 10.1007/s00280-008-0707-9

[CIT0041] Tian M, Neil JR, Schiemann WP. (2011). Transforming growth factor-β and the hallmarks of cancer. Cell Signal 23:951–62. 20940046 10.1016/j.cellsig.2010.10.015 PMC3076078

[CIT0042] Todorova M, Agache V, Mortazavi O, et al. (2013). Antitumor effects of combining metronomic chemotherapy with the antivascular action of ultrasound stimulated microbubbles. Int J Cancer 132:2956–66. 23225339 10.1002/ijc.27977

[CIT0043] Tzu-Yin W, Wilson KE, Machtaler S, et al. (2013). Ultrasound and microbubble guided drug delivery: mechanistic understanding and clinical implications. Curr Pharm Biotechnol 14:743–52. 24372231 10.2174/1389201014666131226114611 PMC4084724

[CIT0044] van Rhoon GC, Franckena M, Ten Hagen TLM. (2020). A moderate thermal dose is sufficient for effective free and TSL based thermochemotherapy. Adv Drug Deliv Rev 163–164:145–56. 10.1016/j.addr.2020.03.006 32247801

[CIT0045] Wei G, Wang Y, Yang G, et al. (2021). Recent progress in nanomedicine for enhanced cancer chemotherapy. Theranostics 11:6370–92. 33995663 10.7150/thno.57828 PMC8120226

[CIT0046] Xia H, Yang D, He W, et al. (2021). Ultrasound-mediated microbubbles cavitation enhanced chemotherapy of advanced prostate cancer by increasing the permeability of blood-prostate barrier. Transl Oncol 14:101177. 34271256 10.1016/j.tranon.2021.101177 PMC8287239

[CIT0047] Yang Y, Li Q, Guo X, et al. (2020). Mechanisms underlying sonoporation: Interaction between microbubbles and cells. Ultrason Sonochem 67:105096. 32278246 10.1016/j.ultsonch.2020.105096

[CIT0048] Zhang Y, Tang N, Huang L, et al. (2021). Effect of diagnostic ultrasound and microbubble-enhanced chemotherapy on metastasis of rabbit VX2 tumor. Med Phys 48:3927–35. 33774845 10.1002/mp.14867

